# Autologous Platelet Concentrates (APCs) for Hard Tissue Regeneration in Oral Implantology, Sinus Floor Elevation, Peri-Implantitis, Socket Preservation, and Medication-Related Osteonecrosis of the Jaw (MRONJ): A Literature Review

**DOI:** 10.3390/biology11091254

**Published:** 2022-08-23

**Authors:** Eitan Mijiritsky, Haya Drora Assaf, Roni Kolerman, Luca Mangani, Vasilena Ivanova, Stefan Zlatev

**Affiliations:** 1 Head and Neck Maxillofacial Surgery, Tel-Aviv Sourasky Medical Center, Department of Otolaryngology, Sackler Faculty of Medicine, Tel-Aviv University, Tel-Aviv 699350, Israel; 2 Faculty of Dental Medicine, Hebrew University of Jerusalem, Jerusalem 9190401, Israel; 3 Department of Periodontology and Oral Implantology, The Maurice and Gabriela Goldschleger School of Dental Medicine, Tel-Aviv University, Tel-Aviv 6997801, Israel; 4 Department of Translational Medicine and Clinical Science, University of Tor Vegata, 00133 Rome, Italy; 5 Oral Surgery Department, Faculty of Dental Medicine, Medical University of Plovdiv, 4000 Plovdiv, Bulgaria; 6 CAD/CAM Center of Dental Medicine at the Research Institute, Medical University-Plovdiv, 4000 Plovdiv, Bulgaria

**Keywords:** PRF, PRP, CGF, autologous platelet concentrates, bone regeneration, dental implants

## Abstract

**Simple Summary:**

Autologous platelet concentrates with high growth factor levels are used in many fields of dentistry. In recent years, the critical role of blood-derived materials in bone and soft tissue engineering has become apparent. After tooth extraction, the alveolar bone is exposed to progressive bone resorption, which can lead to difficulties in implant placement. Hence, many studies have demonstrated that APCs have the potential for soft tissue and bone regeneration. Furthermore, no inflammatory reactions occur, and they may be used alone or in combination with bone grafts, promoting bone growth and maturation. Moreover, the released growth factors and the presence of fibrin structures can induce osteogenesis. This review aims to provide information regarding the applications, indications, advantages, and disadvantages of three APC techniques in hard tissue regeneration.

**Abstract:**

Over recent years, the usage of autologous platelet concentrates (APCs) has risen in hard tissue regeneration and oral implantology. The purpose of the present review is to offer an overview of the use of three APC techniques in dentistry: platelet-rich plasma (PRP), platelet-rich fibrin (PRF), and concentrated growth factor (CGF). A narrative summary of articles published between January 2011 and April 2022 is provided. The PubMed, Cochrane Library, Scopus, and Embase databases were used to conduct the search. The following keywords were used in the preliminary: “VEGF”, “TGF-b1”, “PRP”, “PRF”, “CGF”, AND “sinus augmentation” OR “implants” OR “peri-implantitis” OR “socket preservation” OR “MRONJ”. A total of 82 articles was finally included. The review then takes into account the application of the three techniques in different areas of treatment—including oral implantology, sinus floor elevation, peri-implantitis, socket preservation, and medication-related osteonecrosis of the jaw (MRONJ)—as well as their advantages and disadvantages.

## 1. Introduction

Platelet concentrates have established their role in regenerative medicine in recent years. In a relevant systematic review published by Mijiritsky et al. [1], the authors focused on the application of autologous platelet concentrates (APCs) in soft tissue healing and regeneration. Platelets, which contain growth factors, play significant roles in cell migration, proliferation, differentiation, and angiogenesis, and are associated with tissue regeneration. The centrifugation of venous blood produces autologous platelet concentrates (APCs) at different speeds, along with the use or non-use of thrombin and anticoagulants. The fibrin clot formed after this process contains platelets and leukocytes. There are several generations of APCs. Among them, platelet-rich plasma (PRP), platelet-rich fibrin (PRF), and concentrated growth factor (CGF) can play significant roles. In an article by Qiao et al. [2], the authors revealed that platelet concentrates include growth factors (GFs) such as basic fibroblast growth factor (bFGF), vascular endothelial growth factor (VEGF), insulin-like growth factor 1 (IGF-1), transforming growth factor β-1 (TGF-β1,) and platelet-derived growth factor-BB (PDGF-BB). As a result, they can induce angiogenesis and promote the proliferation and differentiation of osteoblasts. There are established differences regarding the amounts of GFs released using the three different APC techniques (CGF, PRF, and PRP). Using PRF or CGF results in a significant increase in GFs and levels of bFGF during the procedure compared to PRP. However, the levels of the other aforementioned growth factors do not show a marked contrast between the different APCs [2]. The use of APCs in the disciplines presented below is of utmost importance to the scientific community. Our first article discussed the three APCs’ potential in periodontal regeneration and facial rejuvenation. The present article mainly discusses the application of APCs in hard tissue regeneration—i.e., dental implants, sinus floor elevation, socket preservation, medication-related osteonecrosis of the jaw (MRONJ), and peri-implantitis—as well as the advantages and limitations of each technique.

### 1.1. Dental Implants and Sinus Floor Elevation

Replacement of missing teeth with dental implants in the posterior area of the alveolar ridge can lead to challenges due to severe vertical and transversal bone resorption and maxillary sinus pneumatisation. Sinus floor elevation procedures commonly rehabilitate the bone dimensions before implant placement [3]. A transantral lateral approach is performed by fenestration of the anterior sinus wall and elevation of the sinus membrane. This is indicated in cases where the available bone height is insufficient and larger augmentations are needed [4]. The transcrestal maxillary sinus floor elevation is performed to increase the available bone in the vertical dimension in the distal area of the edentulous maxilla. The surgical access is accomplished through the bone crest [5]. The surgical treatment is based on anatomy, sinus health, desired bone augmentation, bone dimensions, general health status, smoking, and oral hygiene. Various grafting materials are employed for sinus lift procedures. Scientific evidence indicates the successful application of deproteinised bovine bone mineral [5], β-TCP [6], freeze-dried bone allografts [7], xenografts [8,9], BMP, stem cells [10], and APCs during sinus lift procedures.

This review discusses the current applications of autologous platelet concentrates for bone regeneration, and focuses on the properties of platelet derivatives compared to other grafting materials in regenerative medicine.

### 1.2. Peri-Implantitis

Berglundh et al. [11] defined the cases of good peri-implant health, peri-implant mucositis, and peri-implantitis in day-to-day clinical practice. A plaque-associated pathological condition occurring in tissues around osseointegrated dental implants, characterised by inflammation in the peri-implant mucosa and subsequent progressive loss of supporting bone, is defined as peri-implantitis. The clinical signs of peri-implantitis include bleeding on probing and/or suppuration on gentle probing, the recession of the mucosal margin, and bone loss in the radiographic examination.

The main purpose of managing peri-implant infections is to remove and eliminate the bacterial biofilm. Some of the nonsurgical (conservative) therapies include mechanical debridement [12,13,14], laser therapy [15,16,17], erythritol air polishing [18], and drug therapy [19,20,21,22]. Conservative therapy alone may not be sufficient to resolve the inflammation process. In order to remove the granulation tissue and the contamination of the implant surface, it is recommended to perform access surgery. Surgical treatment is divided into resective (i.e., bone recontouring) [23,24,25] and regenerative therapy. The regenerative therapy enables optimal peri-implant conditions and eliminates aetiological causes [26]. Various grafting materials and membranes are considered when reconstructive peri-implant surgery is indicated [27].

This review aims to reveal the applications and incorporation of APCs as a treatment modality in cases of peri-implantitis around osseointegrated dental implants.

### 1.3. Socket Preservation

After tooth loss, the alveolar bone undergoes resorption in the vertical and horizontal dimensions. As a result of the bone remodelling, the subsequent implant placement and prosthetic treatment become challenging. Alveolar socket preservation is a surgical procedure that aims to decrease bone resorption and soft tissue collapse after tooth extraction, and leads to improved aesthetic and functional prosthodontic results [28]. Schropp et al. [29] reported that 30% of the alveolar bone width is reduced during the first three months after tooth extraction, and around 50% within the first year. Unequal resorption of the buccal plate compared to the palatal/lingual plate of the ridge has been reported, with the buccal plate undergoing significantly more resorption [30]. Clavero et al. reported that the successful rehabilitation of the edentulous ridge with implants depends on the following parameters: sufficient bone quality and volume, and proper implant position for prosthetics. Therefore, the more significant the amount of bone preserved after tooth extraction, the greater the chances of successful implantation and rehabilitation [31]. A wide variety of grafting materials for socket and bone preservation are documented in the scientific literature. The present review focuses on the benefits of APCs in this procedure.

### 1.4. MRONJ

In 2014 the American Association of Oral and Maxillofacial Surgeons presented the pathology medication-related osteonecrosis of the jaw (MRONJ), as opposed to bisphosphonate-related osteonecrosis of the jaw (BRONJ), due to the rising number of osteonecrosis cases involving the upper and lower jaw, associated with antiresorptive and antiangiogenic agents [32]. MRONJ is defined by the presence of an avascular area of necrotic bone in the maxillofacial area, with or without exposed bone, that has been present for more than eight weeks in a patient who has no oral cancer or previous radiation therapy in this area of the jaw [32,33,34,35].

The management of patients with MRONJ is complexand includes a conservative and surgical approach. The main purpose of the treatment is to decrease the pain and the progression of the infection, and to improve the patients’ quality of life [33]. In 2022, the AAOMS updated their position paper and referred to the use of adipose- or bone marrow-derived-MSCs and PRP for the treatment or prevention of MRONJ in mice. Furthermore, the authors referred to articles by Mozatti et al. and Adornato et al. about the use of APC therapies as a treatment in cases of MRONJ [36]. This review discusses the use of APCs in dental implantation, sinus augmentation, socket preservation, peri-implantitis, and MRONJ, considering the advantages and disadvantages of each technique.

## 2. Materials and Methods

**Figure 1:**
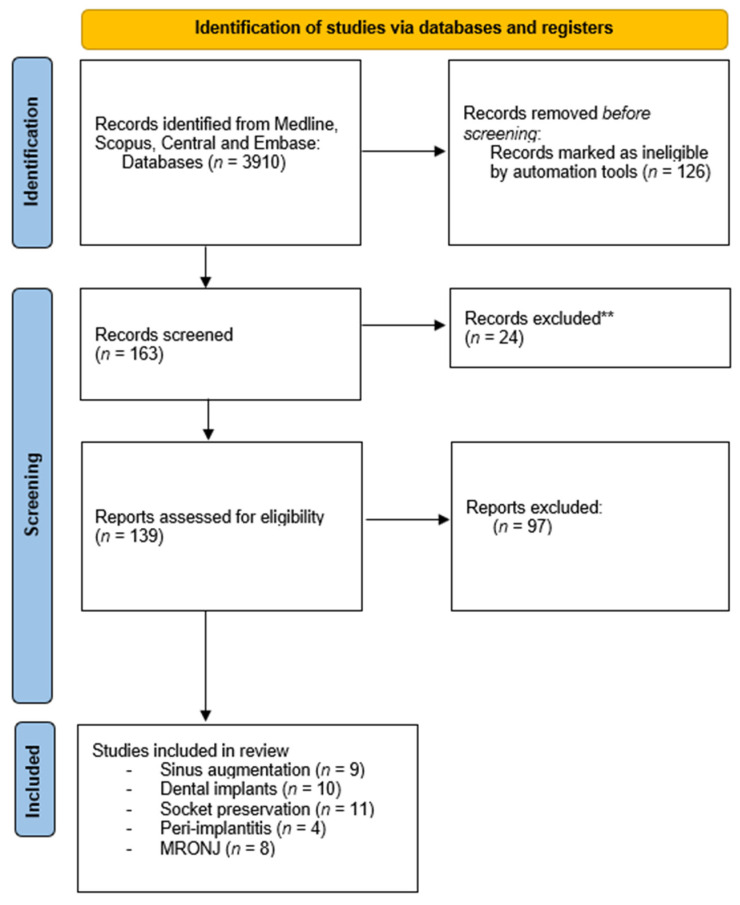
Flowchart of the search strategy.

The PubMed, Cochrane Library, Scopus, and Embase databases were searched from January 2011 to April 2022 to find published studies on the effects of different autologous platelet concentrates on dental implants, sinus floor elevation, peri-implantitis, socket preservation, and MRONJ. (Figure 1) The used keywords were “PRP”, “PRF”, “VEGF”, “CGF”,”TGF-b1”, AND “sinus augmentation” OR “implants” OR “peri-implantitis” OR “socket preservation”. All studies presented in English that investigated the effects of autologous platelet concentrates on the fields mentioned above were considered in the selection process. The review process, including search and selection (i.e., identification, screening, eligibility of included studies), was performed according to the PRISMA criteria. Two independent researchers initially read the titles and the abstracts to identify potentially eligible full-text papers. All authors participated in the selection process for the full-text review, and any conflicts were resolved through discussion. Priority was given to RCT articles—most of them from recent years. Several criteria, listed below, were considered.

### 2.1. APCs in Dental Implants and Sinus Elevation

#### 2.1.1. Inclusion Criteria

- Study design: Randomised controlled trials (RCTs), cohort studies, and cross-sectional studies;
- Population: Human studies, with a minimum of 10 patients and no restriction in terms of patient ages;
- Intervention: Dental implant surgery on patients; only articles with a control group and with data about the follow-up period were included.

#### 2.1.2. General Exclusion Criteria

- Lack of baseline data prior to surgery;
- Patients with systemic diseases or craniofacial anomalies;
- Follow-up of less than 6 months.

### 2.2. APCs in Peri-Implantitis, Socket Preservation, and MRONJ

#### 2.2.1. Inclusion Criteria

- Study design: Randomised controlled trials (RCTs), cohort studies, cross-sectional studies, case reports, and case series in the English language;
- Population: Only studies on humans were included;
- Intervention: Peri-implantitis, socket preservation, and MRONJ treatments for patients; only articles with a control group and with data about the follow-up period were included;
- Types of outcome: Clinical or histological evaluation.

#### 2.2.2. Exclusion Criteria

- Review articles.

A manual search of the references of the selected articles was performed to identify additional publications.

## 3. Results

### 3.1. Dental Implants

The clinical studies regarding the application of PRP, PRF, and CGF in dental implantology that were included in the present review are presented in Table 1. Hartlev et al. [37] compared implants placed in sites that were previously augmented with autogenous bone grafts covered by a PRF membrane (PRF group) or autogenous bone grafts with deproteinised bovine bone mineral and a resorbable collagen membrane (control group). The total number of implants in the PRF group was 14, and in the control group it was 13. There were no statistical differences in implant survival rates or implant crown survival between the groups (implant survival: PRF 100%, control 85%; implant crown survival: PRF 100%, control 92%). Twenty-four months after crown placement, patients were recalled for a follow-up. The radiographic peri-implant marginal bone change at follow-up was 0.26 in the PRF group and 0.68 in the control group. A significant difference was observed between the groups. Both groups demonstrated similarly healthy peri-implant soft tissue values at the follow-up. There were no significant differences in bleeding on probing, probing depth, plaque control record, the width of keratinised tissue, or recession between the groups. Due to the similar outcomes, both approaches can be used for bone augmentation. Pichotano et al. [38] investigated the effectiveness of adding leukocytes and PRF (L-PRF) to deproteinised bovine bone mineral (DBBM) after maxillary sinus augmentation for early implant placement. After the sinus elevation, patients were divided into the DBBM (control) and DBBM + PRF groups. The implants were placed in the control group after eight months and in the PRF group after four months. CBCT showed no significant differences in the graft volume between the groups. The percentage of the new bone formation was increased in the L-PRF group compared to the control group.

Immediately after implantation, the residual graft amount and the implant stability quotient were significantly higher in controls than in the PRF group. The authors concluded that adding PRF to DBBM allowed earlier implant placement (4 months, versus 8 months of healing in the control group) and increased new bone formation. Tabrizi et al. [39] examined implant stability during the healing period after implantation. Twenty patients with missing teeth in the distal area of the maxilla requiring bilateral implants were included and divided into PRF and control groups. Implant stability was measured by resonance frequency analysis at 2, 4, and 6 weeks after implantation, and implant stability quotients (ISQs) were calculated. The mean ISQ was significantly increased in the PRF group compared to the control group 2, 4, and 6 weeks after implantation. The authors concluded that PRF use during the healing period might enhance implant stability. Another study by Boora et al. [40] discussed the effect of PRF on peri-implant tissue responses for three months following one-stage implant placement in the maxillary aesthetic area. The patients were divided into a PRF group and a control group. In 3 months, there was a decrease in probing depth in both groups. There were no significant changes in probing depth or bleeding in either group after 1 and 3 months. The marginal bone level changes had a statistically significantly lower mean value in the PRF group. In order to examine whether PRP has a positive influence on the long-term survival and success of dental implants when used in combination with maxillary sinus augmentation, Attia et al. [41] reinvestigated 37 patients 13 years after implantation (total 210 implants; PRP group: 102 implants, control group: 108 implants). The authors evaluated clinical and radiological outcomes in terms of the long-term effects of PRP on bone healing after sinus lift surgery using an autologous iliac crest bone graft. The two groups did not differ significantly in the parameters. Furthermore, the overall evaluation showed no positive effect of PRP on the results.

Kundu et al. [42] evaluated the effects of PRP and different implant surface topographies on the stability of implants that were immediately loaded. The patients were divided into Group I (without PRP) and Group II (with PRP, where implants were placed after dipping in activated PRP). The authors concluded that PRP had no statistically significant effect on bone height changes. However, a synergistic effect of PRP and square-threaded implants was observed in improved implant stability and bone levels.

Dai et al. [43] aimed to evaluate the efficacy of CGF + mineralised collagen (MC) in the guided bone regeneration (GBR) technique as compared to MC alone. Implants were inserted simultaneously, and CBCT was examined immediately after the operation, and at three and six months. The authors indicated benefits to the CGF + MC group in terms of rapid relief from discomfort after the operation and reduced swelling 2–5 days after the operation compared to the control group. Furthermore, they reported pain reduction on days 2–5 and less need for analgesics on day 2 compared to the control group. There was new bone formation in both groups. However, the buccal plate width was thicker in the CGF group three and six months after the operation.

Chen et al. [44] evaluated the clinical and radiological results of modified osteotome sinus floor elevation with CGF and simultaneous short implant placement in cases with residual bone height (RBH) of 2–4 mm in the severely atrophic maxilla. A significant vertical bone reduction was observed in the first six months after the operation. The mean RBH after 12 months was 9.40 ± 0.47 mm, and 100% implant survival was observed. Koyuncu et al. [45], in their preliminary study, evaluated the effects of CGF on implant stability using an RFA device during the early healing period (up to four weeks after implantation). Twelve patients who required dental implants in the anterior mandible participated. One socket was prepared conventionally (control group), while the other was covered with a CGF membrane. No significant difference in ISQ values was measured between the groups. The authors concluded that CGF had a neutral effect on osseointegration compared to the control group.

In contrast to the study above, Pirpir et al. [46] found that CGF positively affected implant stabilisation. In their study, there was a significant difference in ISQ values in the CGF group compared to the control group, and the ISQ values were significantly increased in the CGF group one and four weeks after implantation compared to the control group.

### 3.2. Sinus Augmentation

The clinical studies regarding the application of PRP, PRF, and CGF in sinus augmentation procedures are presented in Table 2. Stumbras et al. [47] compared bone regeneration in the anterior maxilla between bone substitutes and autologous platelet concentrates after alveolar ridge preservation. The study was conducted on 40 patients requiring tooth extraction, who were randomly allocated into three groups: bovine bone mineral covered with resorbable native collagen membrane (BBM/CM), freeze-dried bone allografts covered with resorbable native collagen membrane (FDBA/CM), and plasma rich in growth factors (PRGF) alone. The histomorphometric analysis revealed that the PRGF group was associated with the highest new bone mineral formation.

In their retrospective study, Zhang et al. [48] assessed the influence of PRF on bone regeneration in sinus augmentation procedures in combination with deproteinised bovine bone mineral. Eleven sinuses from ten patients with posterior maxillary bone atrophy were divided into two groups. In the test group, six sinus floor elevations were grafted with a Bio-Oss and PRF mixture, and in the control group, five sinuses were treated with Bio-Oss alone. The authors reported a similar composition and distribution of histological structures for the PRF and control groups six months after the sinus elevation procedures.

Simonpieri et al. [49] evaluated the effectiveness of using leukocyte PRF (L-PRF) in sinus elevation procedures and implantation as the sole grafting material. All patients were followed up for a minimum of 2 years. The authors reported vertical bone gain between 8.5 and 12 mm. L-PRF as the sole grafting material during simultaneous sinus lift and implantation seemed to be a reliable surgical option, promoting natural bone regeneration. Hence, using L-PRF as the sole grafting material during simultaneous sinus elevation is a viable bone regeneration option.

Another retrospective study by Aoki et al. [50] aimed to evaluate the application of PRF alone in sinus lift procedures. Seventy-one implants were placed three months after the sinus elevation in the thirty-four patients included in the study. After five years of follow-up, the cumulative survival rate was 85.5% (implant-based analysis).

In the study by Olgun et al. [51], the authors clinically, radiologically, and histologically compared the effects of T-PRF material alone on bone formation to those of allografts in a two-stage sinus floor elevation. Eighteen participants with a diagnosed residual crest height of less than 5 mm in the posterior maxilla were included. Ten sinuses were randomly assigned to T-PRF as the test group, and eight were assigned to allografts as the control group. The average ISQ values of the L-PRF and control groups were 68.50 and 66.37, respectively. The rate of newly formed bone in the allograft group was not significantly higher than that in the T-PRF group. The authors concluded that bone formation in the T-PRF group accelerated more rapidly than in the allograft group.

Tatullo et al. [52] clinically and histologically evaluated the reconstructive potential of PRF combined with deproteinised bovine bone mineral (Bio-Oss), in comparison with a control group in which only deproteinised bovine bone mineral (Bio-Oss) was used, as grafting materials in pre-implantology sinus grafting of severe maxillary atrophy. The results of the study revealed that using PRF reduces the healing time. The primary stability of implants was observed at 106 days, compared to 120–150 days in the literature. Histological evaluation revealed that PRF induced remarkable neoangiogenesis compared to the control group.

Khairy et al. [53] evaluated the bone quality after sinus augmentation with autogenous bone with or without PRP. Application of PRP did not significantly improve bone density or morphometric value at three months post-grafting. The group without PRP showed statistically significantly higher mean bone density (MBD) immediately after implant insertion and six months after implantation. In their study, Nizam et al. [54] performed 26 maxillary sinus augmentation procedures using a DBBM and L-PRF mixture (test) or DBBM alone (control), in a split-mouth design. Implants were placed and loaded six months after augmentation. The authors reported no significant differences between the groups in histological, histomorphometric, and radiographic evaluations. In both groups, the new bone was in contact with the residual grafting material.

### 3.3. Socket Preservation

#### 3.3.1. PRP

Célio-Mariano et al. [55] radiographically evaluated the effects of PRP after the bilateral extraction of impacted mandibular third molars. The study’s control group was one side left with a blood clot, whereas the test group included the other post-extraction side, which was filled with PRP. In sockets treated with PRP, bone formation was significantly accelerated 1–3 months after extraction compared to the control group.

In a prospective study, Aftab et al. [56] investigated the effects of autologous PRP gel on healing, postoperative pain, facial swelling, trismus, and bone regeneration potential after the surgical removal of impacted mandibular third molars. The study was conducted on 100 patients randomly allocated into experimental (PRP) and control (blood clot) groups. The results revealed significantly decreased postoperative pain, reduced facial swelling, and improved interincisal opening in the PRP group compared to the control group. A comparison of trabecular patterns and bone density revealed that by 10 and 16 weeks postoperatively, significant differences were found in the PRP group compared to the control (Table 3).

#### 3.3.2. PRF

A study by Ivanova et al. [57] compared the effectiveness of socket preservation procedures with freeze-dried bone allograft in combination with PRF, PRF as the sole grafting material, and a control group, which was left with a blood clot only. The PRF group showed significantly increased bone formation compared to the control group. In another study, Canellas et al. [58] evaluated the effect of L-PRF as a sole grafting material after extraction, and it was compared to a control group. Histomorphometric analysis revealed a higher percentage of new bone formation in the L-PRF group after three months. Yelameli et al. [59] compared the effectiveness of PRF and PRP on soft and hard tissue healing after third-molar extraction. Each patient suffered from bilateral third molar impaction, and one socket was filled with PRF and the other with PRP. Four months postoperatively, the mean values of bone density were significantly increased in the PRF group compared to the PRP group. Rao et al. [60] were also interested in investigating the effects of autologous platelet-rich fibrin gel on bone regeneration following bilateral third-molar extraction. The radiological bone filling was reported with higher mean pixels in PRF cases than controls, both immediately and 1, 3, and 6 months post-operation, with no significant differences between the groups.

Contrary to the articles above, Baslarli et al. [61] reported that PRF might not enhance bone healing in impacted mandibular third-molar extraction sockets 30 and 90 days after surgery. Kumar et al. [62] compared the effectiveness of PRF to a control group after third-molar extraction. The authors found that the application of PRF significantly decreased the severity of immediate postoperative sequelae and reduced preoperative pocket depth. Bone density was higher in the case group than in the control group, but this difference was not statistically significant. Bilginaylar et al. [63] evaluated the influence of PRF on pain reduction and consumption of analgesics. They found a significant reduction in pain on days 1–3 postoperatively in the PRF group, and there was also a significant reduction in the control group. Marenzi et al. [64] also reported positive effects of L-PRF in managing postoperative pain, enhancing soft tissue healing, and reducing inflammation after extractions.

### 3.4. Peri-Implantitis

#### 3.4.1. PRP

In a study by Wishnu et al., the authors evaluated the applicability of platelet-rich plasma to enhance bone and soft tissue healing around single-piece implants subjected to immediate loading. They compared this with a control site not treated with PRP. The results revealed that the average bone loss was higher in the group without PRP 3–12 months postoperatively. There were no significant differences in mobility and peri-implantitis [65] (Table 4).

#### 3.4.2. PRF

Hamzacebi et al. [66] examined the clinical effectiveness of PRF application during conventional flap surgery for the treatment of peri-implant bone loss in comparison to a control group (flap surgery with no PRF). Three months after the operation, the mean probing depth was decreased in the PRF group. Moreover, at 3 and 6 months, the CAL (clinical attachment level) was increased in the PRF group compared to the control group, and the keratinised mucosa was significantly increased in the PRF group.

Sun et al. [67] examined the viability of PRF combined with guided bone regeneration (GBR) in the reconstruction of peri-implantitis bone defects. In the control group, patients were treated with flap curettage combined with GBR. The authors reported that the regenerated bone density of the observation group was significantly higher at both 60 days and 120 days after surgery. Furthermore, the pain 24 h after the manipulation was milder in the PRF group.

#### 3.4.3. CGF

Isler et al. [68] examined bone substitutes’ clinical and radiographic effectiveness combined with concentrated growth factor (CGF) or collagen membranes for regenerative surgical treatment of peri-implantitis. Six and twelve months after surgery, significant decreases were observed in the mean gingival index (GI), bleeding on probing (BOP), probing depth (PD), clinical attachment level (CAL), and mucosal recession (MR) values for both groups. The authors concluded that CGF, as well as collagen membranes, yielded successful outcomes.

### 3.5. MRONJ

#### 3.5.1. PRP

Longo et al. [69] evaluated the use of PRP in patients with BRONJ after using alendronate, pamidronate, and zoledronic acid (duration of use: 4–62 months). The surgery without PRP group showed a lower success percentage (53%) than the PRP group (94%). Nevertheless, the two groups showed no significant differences in successful outcomes during the different stages (Table 5).

**Table 3:** Comparison between the 3 APC techniques (PRP, PRF, and CGF) in socket preservation.

Year	First Author	Objectives	Methods	Results (mm)	Authors’ Conclusions
2012	Célio-Mariano et al. [55]	Radiographic evaluation of bone regeneration after application of PRP in tooth sockets.	Thirty bilateral impacted mandibular third molars were divided into a test group (post-extraction socket filled with PRF) and control group.	Increased bone formation in the test group (*p* < 0.01) in the first month (*p* < 0.01), second month (*p* < 0.05), and third month (*p* < 0.01).	Autologous PRP accelerates alveolar bone regeneration after open tooth extraction.
2020	Aftab et al. [56]	Efficacy of socket preservation with autologous PRP gel after surgical extraction of the impacted mandibular third molar.	One hundred patients were allocated into two groups. PRP gel was placed in the extraction socket after extraction in the experimental group, while the control group was without PRP gel.	The test group had significantly lower pain scores, reduced facial swelling, and improved interincisal opening. Significant differences (*p* < 0.005) were observed between the two groups radiographically at the 10th and 16th weeks.	Application of autologous PRP gel may enhance the wound-healing process and promote bone regeneration.
2021	Ivanova et al. [57]	Randomised controlled clinical trial to evaluate the efficacy of A-PRF alone or in combination with freeze-dried bone allografts in improving vital bone formation and decreasing alveolar bone resorption.	Sixty patients with sixty-three post-extraction sockets were divided into three groups.	Bone resorption was less pronounced in both tested groups than in the control group, where these values were significantly higher.	The study demonstrated a novel in vivo method for measuring bone resorption after ridge augmentation procedures.
2020	Canellas et al. [58]	A prospective, single-blind, parallel, randomised, controlled clinical trial to evaluate the efficacy of leukocyte- and platelet-rich fibrin (L-PRF) in socket preservation after tooth extraction.	Forty-eight subjects for non-molar tooth extraction randomly assigned to the L-PRF group (*n* = 24) or the control group (*n* = 24). Cone-beam computed tomography was performed immediately after tooth extraction and three months after tooth extraction, prior to implant surgery.	A significant difference in bone resorption was registered 1 mm below the crest: 0.93 ± 0.9 mm for the L-PRF group and 2.27 ± 1.2 mm for the control group (*p* = 0.0001). New bone formation in the L-PRF group was compared with the control group; the values were 55.96 ± 11.97% and 39.69 ± 11.13%, respectively (*p* = 0.00001).	The administration of L-PRF should always be considered when socket preservation is planned.
2015	Yelameli et al. [59]	Comparison of the utility and effectiveness of platelet-rich fibrin (PRF) with that of platelet-rich plasma (PRP) in soft tissue healing and bone tissue healing of extracted third molar sockets.	Split mouths of 20 patients underwent bilateral extraction of impacted third molars	Soft tissue healing recorded at one week post-operation for the PRF group was significantly higher than that for the PRP group.	PRF is significantly better than PRP in promoting soft tissue healing and faster regeneration of bone after third-molar extraction.
2013	Rao et al. [60]	Autologous platelet-rich fibrin gel (PRF gel) for bone regeneration following extraction.	Forty-four bilateral mandibular third molars were divided into a test group (PRF gel) and control group.	Follow-up on the first postoperative day, the first week, and one month, three months, and six months post-operation revealed no statistically significant differences between the groups	Further follow-up of the present patients and a larger sample size are required to obtain conclusive results of the bone regeneration in extraction sockets with PRF gel.
2015	Baslarli et al. [61]	Clinical and radiological assessment of extraction sockets filled with PRF.	Forty bilateral impacted mandibular third molars were extracted from twenty patients. The test group consisted of post-extraction sockets filled with PRF, while the control group had a blood clot only.	No statistically significant differences between PRF-treated and non-PRF-treated sockets 30 and 90 days post-operation.	More research is needed to support the advantages of PRF in tissue regeneration.
2015	Kumar et al. [62]	Clinical and radiological assessment of the effects of PRF after third-molar extraction.	Thirty-one patients were included and divided into a test group (PRF placed in the post-extraction socket) and control group. Pain, swelling, maximum mouth opening, periodontal pocket depth, and bone formation were evaluated, with a follow-up period of 3 months.	Significant differences between the control and test groups with regard to the evaluated parameters.	The application of PRF decreases the postoperative pain and swelling, decreases preoperative pocket depth, and induces bone formation.
2016	Bilginlayar et al. [63]	Postoperative outcomes after removing 80 impacted mandibular third molars from 59 patients.	Eighty impacted mandibular molars were divided into four groups: a control group (with conventional burs), second group (PRF), third group (piezosurgery), and fourth group (piezosurgery and PRF placed in the post-extraction socket).	Significant reduction (*p* < 0.05) in pain on days 1, 2, and 3, and in the number of analgesics taken on days 2 and 3, in both PRF groups.	There were no significant differences in swelling or trismus between the control group and the other groups.
2015	Marenzi et al. [64]	Effects of leukocyte- and platelet-rich fibrin (L-PRF) on pain and soft tissue healing after tooth extractions.	Clinical evaluation of 108 tooth extractions performed on 26 patients divided into test and control groups.	After 7 days, modified healing index values in the experimental and control groups were 4.8 ± 0.6 and 5.1 ± 0.9, respectively.	The application of L-PRF in post-extraction sockets is a useful procedure to manage postoperative pain and to promote the soft tissue healing process, reducing the early adverse effects of the inflammation.

**Table 4:** Comparison between the 3 APC techniques (PRP, PRF, and CGF) in peri-implantitis.

Year	First Author	Objectives	Methods	Results (mm)	Authors’ Conclusions
			PRP		
2019	Vishnu et al. [65]	Platelet-rich plasma to enhance osseous and associated soft tissue healing around single-piece implants was subjected to immediate loading and compared with a control site not treated with PRP.	Twenty completely edentulous patients were selected, and two one-piece implants were placed for mandibular overdenture.	There was less marginal bone loss, probing depth, percussion, implant mobility, and peri-implantitis around implants treated with PRP.	PRP can be used as a viable treatment adjunct in immediately loaded one-piece implants.
			PRF		
2015	Hamzacebi et al. [66]	Clinical effectiveness of the application of platelet-rich fibrin (PRF) and conventional flap surgery to treat peri-implant bone loss.	Nineteen patients with peri-implant bone loss were randomly allocated to the PRF test group or the control group with only an access flap.	The increase in keratinised mucosa from baseline to 6 months post-operation was statistically significant for the PRF group (*p* < 0.001).	The application of PRF in peri-implant bone loss provided better clinical results than conventional flap surgery.
2021	Sun et al. [67]	Clinical effects of platelet-rich fibrin (PRF) in guided bone regeneration (GBR) for peri-implantitis bone defects.	Eighty patients were divided into two groups. The control group included patients treated with GBR and flap curettage, and the observation group included patients treated with PRF and bone powder.	Significantly higher bone density in the observation group after 120 days (*p* < 0.001). Postoperative complaints were milder in the PRF group.	The combination of PRF and GBR has a noticeable effect in repairing bone defects in patients with peri-implantitis, and can reduce patients’ pain during the healing period.
			CGF		
2018	Isler et al. [68]	Evaluation of the clinical and radiographic results after regenerative surgical treatment (RST) of peri-implantitis with collagen membranes (CMs) or concentrated growth factor (CGF) during 12-month follow-up.	Clinical assessment was conducted on 52 patients with peri-implantitis lesions at baseline, and at 6 and 12 months after the operation.	No statistically significant differences were observed in the clinical parameters between the two groups after six months.	Using a collagen membrane in combination with a bone substitute showed better results at 12 months in RST of peri-implantitis.

**Table 5:** Comparison between the 3 APC techniques (PRP, PRF, and CGF) in MRONJ.

Year	First Author	Objectives	Methods	Results (mm)	Authors’ Conclusions
2014	Longo et al. [69]	Use of bisphosphonates (BPs) to treat bone metastases and various bone diseases.	Seventy-two patients with BRONJ with nonsurgical therapy, surgical therapy, and surgical therapy with platelet-rich plasma (PRP) gel to evaluate the therapeutic effects.	PRP’s good results in improving wound healing provided definitive evidence of its effectiveness.	Recently, it has been proposed to rename BRONJ to antiresorptive-agent-related osteonecrosis of the jaw (ARONJ).
2020	Mauceri et al. [70]	Longitudinal hospital-based study evaluation at two years of a standardised medical–surgical protocol for dental extraction, combined with platelet-rich-plasma (PRP) application, compared with a conventional protocol in cancer (ONC) and osteometabolic (OST) patients at risk of bisphosphonate (BP)-related ONJ.	Of 20 patients, 6 received BPs for skeletal-cancer-related events (34.17 ± 19.97 months), and 14 received BPs for metabolic bone disease (74.5 ± 34.73 months). Patients underwent a standardised protocol for dental extraction combined with PRP.	Success in all patients treated with PRP.	Combining a standardised medical–surgical protocol with the application of PRP may limit the occurrence of BP-related ONJ. in both ONC and OST patients.
2014	Kim et al. [71]	A single-group study using leucocyte-rich and platelet-rich fibrin (L-PRF) for treating bisphosphonate-related osteonecrosis of the jaw (BRONJ).	After treatment with L-PRF, the response of each patient was recorded once per month for four months post-operation. Among the total of 34 patients, 26 (77%) showed complete resolution, 6 (18%) showed delayed resolution, and 2 (6%) showed no resolution.	A significant association between treatment and the stage of BRONJ (*p* = 0.002).	This study showed that it is feasible to use L-PRF for the treatment of BRONJ, but the effectiveness cannot be judged based on this study’s design.
2016	Norholt et al. [72]	Treatment of osteonecrosis of the jaw (ONJ) with additional use of autologous membranes of platelet-rich fibrin (PRF).	Fifteen patients with ONJ lesions in the maxilla (*n* = 3), mandible (*n* = 11), or both (*n* = 1).	Follow-up 7–20 months after surgery; complete mucosal healing and absence of symptoms were found in 14 of the 15 patients (93%).	The use of PRF membranes in the surgical treatment of grade 2 ONJ may contribute to successful outcomes.
2016	Park et al. [73]	Comparison of the healing outcomes of combined use of BMP-2 and L-PRF with those of a single use of L-PRF for treating medication-related osteonecrosis of the jaw (MRONJ).	Of 55 patients with MRONJ, 25 were treated with L-PRF alone, and 30 were treated with L-PRF and recombinant human BMP-2.	Surgical sites were evaluated postoperatively at 4 and 16 weeks, and showed more favourable outcomes with complete resolution of the lesions compared with therapy using L-PRF alone (*p* = 0.028).	The combined use of BMP-2 and L-PRF leads to the early resolution of MRONJ.
2018	Giudice et al. [74]	Efficacy of platelet-rich fibrin (PRF) after bone surgery compared to surgery alone for osteonecrosis of the jaw (MRONJ).	Forty-seven patients with a diagnosis of stage II or III MRONJ were allocated to two groups.	Analysis of mucosal integrity, absence of infection, and pain evaluation showed a significant difference in PRF only at T1 (*p* < 0.05).	Local application of PRF after bone surgery may improve the quality of life during the short-term follow-up and reduce pain and postoperative infections.
2017	Asaka et al. [75]	Effectiveness of platelet-rich fibrin (PRF) as a wound-healing accelerator in patients undergoing oral bisphosphonate therapy and requiring tooth extractions.	One hundred and two patients were divided into a PRF group and a control group.	There were no intraoperative complications in patients with medication-related osteonecrosis of the jaw (MRONJ).	Early epithelisation was confirmed in all PRF patients.

#### 3.5.2. PRF

Mauceri et al. [70] aimed to evaluate the use of PRP in post-extraction sockets in patients with cancer and osteometabolic patients at risk of BRONJ (bisphosphonate-related osteonecrosis of the jaw). The study’s outcomes revealed that two years after extraction and treatment with PRP, no patient had clinical or radiological signs of osteonecrosis of the jaw.

In a single-group study, Kim et al. [71] evaluated the use of PRF in patients treated with bisphosphonates and diagnosed with BRONJ. The authors reported a significant association between the response to treatment and the stage of BRONJ. PRF use yielded complete resolution in 77% of patients; 18% showed delayed resolution, and 6% had no resolution. The latter patients were being treated with zoledronate and chemotherapy.

Norholt et al. [72] evaluated the benefits of PRF application during surgical treatment in cases of osteonecrosis of the jaw (ONJ). Fifteen patients were treated surgically with resection of the necrotic bone of the jaw. At follow-up 7–20 months after surgery, complete mucosal healing and an absence of symptoms were found in 93% of the patients.

Park et al. [73] investigated the additional use of bone morphogenetic protein 2 (BMP-2) with PRF to treat MRONJ patients. At 4 and 16 weeks post-operation, patients treated with both L-PRF and BMP-2 showed favourable outcomes, with complete resolution of the lesions, which was statistically significant compared with the therapy using L-PRF alone. The authors concluded that further use of BMP-2 improved the healing.

Another study by Giudice et al. [74] evaluated the efficacy of PRF after bone surgery compared to surgery alone in the treatment of MRONJ. The results revealed that one month after surgery, the use of PRF yielded a significant decrease in VAS scores compared to the control group. After a follow-up of one year, the authors reported that the mucosal integrity was also superior in the PRF group (95.8% and 91.3%, respectively).

Asaka et al. [75] evaluated the use of PRF in dental extraction in MRONJ patients. Early epithelisation was observed in the PRF group compared to the control group (2–4 and 2–8 weeks, respectively).

## 4. Discussion

The aim of this narrative review was to summarise the information regarding the indications, advantages, and disadvantages of autologous platelet concentrates applied in bone regeneration procedures, including sinus augmentation, dental implants, socket preservation, peri-implantitis, and MRONJ. As Mijiritsky et al. [1] described, the PRP, PRF, and CGF techniques differ in the amounts of GFs, induction of angiogenesis, and preparation techniques. CGF and PRF contain significant amounts of GFs, and are more capable of inducing angiogenesis than PRP. Hence, wound healing increases in the former two techniques compared to PRP. Furthermore, the preparation of PRP requires two stages of centrifugation, while the other two techniques require only one stage.

There are references to the leading articles in the categories mentioned above in the present review—most of them from recent years. In 1982, Branemark was the first to bring dental implants to North America. [76] Since that time, dental implants have provided a successful way to rehabilitate edentulous ridges.

Raghavendra et al. [77] described the changeover from mechanical stability to biological stability in implants during the process of osseointegration. While primary stability is achieved upon implant placement, secondary stability depends on the new bone formation. Various strategies and materials have been used to accelerate osseointegration, including biologically active molecules that induce osteoblastic differentiation, osteoconductivity, peri-implant bone healing, BMP, and growth factors [78,79]. The present article reviewed and compared the three APC techniques in dental implants. Choukroun et al. [79] revealed that in PRP, the fibrin organisation consists of tetramolecular or bilateral junctions with strong thrombin concentrates. As a result, there is a rigid network, which is not very conducive to cytokine enmeshment and cellular migration.

In contrast, PRF consists of a significant percentage of equilateral junctions, leading to weak thrombin concentrations. As a result, a flexible and fine network enables cytokine enmeshment and cellular migration. In a study by Masuki et al. [80], the authors aimed to compare the growth factor contents in PRP and its derivatives, such as advanced PRF (A-PRF) and concentrated growth factor (CGF). They concluded that both A-PRF and CGF contained significant growth factors, and that they would function as scaffolding materials and as reservoirs to deliver certain growth factors at the application site.

In terms of resorption time, while in PRP the release of GFs takes place within the first day [81], in PRF, there is a slow release of GF and cytokines over 10 days, and in CGF, there is a sustained release of GFs up until 28 days [2]. The rapid versus sustained release of GFs is significant in the delivery of GFs to the target site of implantation.

The present article reviewed the added value of using APCs in early implant placement after sinus augmentation, implant stability, new bone formation, and peri-implant parameters such as plaque index, probing depth, and bleeding index. Research reveals that APCs can promote peri-implant bone regeneration and alleviate the postsurgical clinical experience. Furthermore, Tabrizi et al. [39] conducted a clinical study on 20 patients with missing teeth in the distal area of the maxilla requiring bilateral implants, who were divided into two groups: a PRF group and a control group. Implant stability was measured by resonance frequency analysis at 2, 4, and 6 weeks after implantation, and implant stability quotients (ISQs) were calculated. The mean ISQ was significantly increased in the PRF group compared to the control group. The authors concluded that PRF might increase the biological stability of dental implants placed in the distal areas of the maxilla during the healing period. Pirpir et al. [47] investigated the effects of CGF on implant stability and osseointegration. The authors observed that the concentrated growth factor positively affected implant stabilisation. The ISQ measurements in weeks 1 and 4 were significantly higher in the study group. Another study by Attia et al. [41] evaluated clinical and radiological outcomes in the long-term effects of PRP on bone healing after sinus lift surgery using an autologous iliac crest bone graft. The results did not provide evidence of a positive effect of PRP. Since the data in the literature are controversial with respect to the benefits of APCs in bone regeneration around dental implants, there is a specific need for long-term follow-up studies to assess whether the positive effects of these biological materials persist over time. More laboratory and clinical studies are necessary to establish the advantages of blood products in the osseointegration process.

As previously described [43,54], the application of PRP leads to enhanced implant stability and improved bone density in sinus augmentation procedures. Furthermore, scientific evidence [50,51] indicates that PRF could be applied as a sole grafting material in cases of lower residual bone height. The application of PRF could be considered an effective and safe procedure for sinus elevation and promoting natural bone regeneration with or without simultaneous implant placement. Research shows that PRF, in combination with different bone graft materials, is beneficial to the clinical, histological, and radiographic outcomes after sinus lift elevation and implant placement [38,49,52,53]. In order to establish these findings, the authors conclude that long-term follow-up and larger samples are necessary.

Management of BRONJ is a controversial topic, in which prevention plays a pivotal role. When conservative treatment cannot result in cure or improvement, surgical intervention for removal of the bone is necessary. Due to the ability of PRP to increase tissue vascularisation and release multiple growth factors, the use of PRP has been suggested by many authors to enhance postsurgical wound healing [70,71]. PRP releases a wide variety of key biological mediators that are important during tissue repair [82]. Research shows that platelet concentrates add growth factors to the surgical site and accelerate the healing of bone and soft tissue [73]. Scholars report that APCs could contribute to the treatment of osteonecrosis by improving patients’ quality of life and reducing pain and postoperative infections [74,75,76]. Nevertheless, conclusive statements regarding the successful outcomes of this choice of treatment material cannot be made. More randomised clinical trials at different stages of the development of BRONJ are necessary to prove this hypothesis.

## 5. Conclusions

This narrative review provides clinical information regarding three APC techniques used in sinus augmentation, dental implants, socket preservation, peri-implantitis, and MRONJ: PRP, PRF, and CGF. An advantage of using APCs is the ability to deliver a substantial quantity of GFs to the target site, thus promoting angiogenesis and wound healing. The most established technique seems to be PRP, which ensures a more rapid delivery of GFs compared to the other two methods. At early times, PRP provides more rapid delivery of GFs to the target site than PRF or CGF. As a result of the addition of PRP to a bone autograft, dense and mature bone is formed. However, the transmission of infectious diseases and coagulopathies is an important limitation to the PRP technique, and should be considered. Conversely, CGF requires only centrifuged autologous blood and, therefore, provides immunological biocompatibility. CGF is used in oral surgery, primarily for hard tissue regeneration.

This narrative review also discusses the three APC techniques in the context of MRONJ. The biggest advantage of using these techniques is the ability to deliver high quantities of GFs to the target site. The use of PRP in surgical treatments was successful. PRF used in surgical treatments promotes significant early epithelisation. CGF as a combination of surgical therapy appears to be a practical approach to improving tissue healing. The local application of CGF seems to be an effective approach to the surgical treatment of MRONJ in osteoporosis patients, improving tissue regeneration. This is a narrative review, which serves as a limitation of this study. Thus, interpretation should be made with caution. Additional high-quality studies should be conducted, e.g., randomised controlled trials. Future systematic reviews and meta-analyses regarding the topic of this review are also warranted.

## Figures and Tables

**Table 1:** Comparison between the 3 APC techniques (PRP, PRF, and CGF) in dental implants.

Year	First Author	Objectives	Methods	Results (mm)	Authors’ Conclusions
			PRP		
2018	Tabrizi et al. [39]	Evaluation of the impact of PRF on implant stability in the distal areas of the upper jaw.	Twenty patients requiring bilateral implants in the distal areas of the maxilla were included and divided into PRF and control groups. Implant stability was assessed by resonance frequency analysis (RFA) at 2, 4, and 6 weeks after placement.	Significant differences in the mean ISQ values were found between the groups at two weeks (*p* = 0.04), four weeks (*p* = 0.014), and six weeks (*p* = 0.027) after placement.	PRF may enhance implant stability during the healing period of implants placed in the posterior maxilla.
2015	Boora et al. [40]	Clinical and radiological evaluation of the effect of PRF on bone and soft tissue structures following one-stage implant placement in the maxillary aesthetic area.	The patients were divided into a PRF group and a control group. The parameters of interest were probing depth and marginal bone level around implants.	In 3 months, there was a decrease in probing depth in both groups. There were no significant changes in probing depth or bleeding in either group after 1 and 3 months. The marginal bone level changes had a statistically significantly lower mean value in the PRF group	PRF may have a beneficial effect on the peri-implant tissues.
2020	Attia et al. [41]	The long-term impact of PRP regarding clinical and radiological outcomes on the inserted implants after maxillary augmentation in the RCT.	Consideration of plaque index, probing depth, bleeding index, mobility grade, Periotest^®^ values, and radiological bone loss.	In 36% of the results, the PRP group was superior to the control group.	The results showed no positive effect of PRP on the clinical and radiological outcomes.
2014	Kundu et al. [42]	Evaluation of the impact of PRP and different implant surface topographies on the stability of implants that were immediately loaded.	The patients were divided into two groups—with or without PRP.	PRP had no statistically significant effect on bone height changes.	The results revealed no significant effect of PRP on bone height. There was an improvement in implant stability in the PRP and square thread-form implant group.
2020	Dai et al. [43]	Clinical evaluation of the effectiveness of concentrated growth factor (CGF) in combination with mineralised collagen (MC) in guided bone regeneration (GBR).	GBR technique with simultaneous implant placement was performed on 29 patients with CGF and MC, or with MC as the sole grafting material. CBCT was examined immediately after the operation, as well as at three and six months.	Benefits to the CGF + MC group in terms of rapid relief from discomfort after the operation and reduced swelling.	Milder clinical symptoms, reduced postoperative discomfort, and increased bone regeneration were observed in the CGF + MC group.
			PRF		
2021	Hartlev et al. [37]	Survival and clinical performance of implants placed in sites previously augmented with autogenous bone grafts covered by PRF membrane (PRF group) or autogenous bone graft with deproteinised bovine bone mineral and a resorbable collagen membrane (control group).	The test group included 14 placed implants, while the control group included 13. Patients were recalled for evaluation 24 months after prosthetic rehabilitation.	The radiographic peri-implant marginal bone change at follow-up was 0.26 in the PRF group and 0.68 in the control group.	Both approaches can be used for bone augmentation. There was an increased marginal bone level in the PRF group compared to the control group.
2018	Pichotano et al. [38]	Evaluation of the impact of leukocyte- and platelet-rich fibrin (L-PRF) added to deproteinised bovine bone mineral (DBBM) for early implant placement after maxillary sinus augmentation.	In a split-mouth design, 12 patients were divided into a test group (DBBM + L-PRF) and a control group (DBBM as the sole grafting material). Implants were placed four months after the augmentation in the test group, and after eight months in the control group.	Primary stability was significantly higher in the control group (75.13 ± 5.69). Newly formed bone was higher in the test group (44.58% ± 13.9%).	Adding PRF to DBBM allowed early implant placement (4 months, versus 8 months of healing in the control group) with increased new bone formation.
2015	Boora et al. [40]	Effect of PRF on peri-implant tissue three months following one-stage implant placement in the maxillary aesthetic area.	Twenty patients were randomly divided into a test group (PRF) group and a control group.	There were no significant changes in probing depth or bleeding in either group after 1 and 3 months.	PRF could be considered a therapeutic s supplement in cases of one -tage, single-tooth implant placement in the aesthetic area of the maxilla.
			CGF		
2016	Chen et al. [44]	Clinical and radiological results of modified osteotome sinus floor elevation (OSFE) with CGF and simultaneous short implant placement in cases with residual bone height (RBH) of 2–4 mm in the severely atrophic maxilla.	Sixteen patients were included in the study. Twenty-five short implants were placed using the modified OSFE with CGF. Vertical bone gain (VBG) was measured using cone-beam computed tomography.	The mean residual bone height 12 months after surgery was 9.40 ± 0.47 mm.	Modified OSFE with CGF application and simultaneous short implant placement resulted in predictable clinical results for severely atrophic maxilla with RBH of 2–4 mm.
2020	Koyuncu et al. [45]	Effect of concentrated growth factor (CGF) on dental implant stability in type 2 bone using the resonance frequency analysis (RFA) device Smartpeg^®^.	The study included 12 patients who required dental implants in the anterior mandible. One socket was prepared conventionally (control group), while the other was covered with a CGF membrane. Implant stability was measured upon implant placement and at the first, second, and fourth weeks.	No statistically significant differences were observed between the ISQ values in either of the groups.	CGF did not benefit dental implant stability in the early healing period in type 2 bone.
2017	Pirpir et al. [46]	The effects of CGF on implant stability and osseointegration.	Twelve patients were divided into a test group (where implant bed was covered with a CGF membrane) and a control group. Implant stability was measured immediately after implant placement and at the first and fourth weeks.	The mean ISQ values were significantly higher in the test group during the period of evaluation.	Concentrated growth factors had positive effects on implant stabilisation. The ISQ measurements in week one and week four were notably higher in the study group.

**Table 2:** Comparison between the 3 APC techniques (PRP, PRF, and CGF) in sinus augmentation.

Year	First Author	Objectives	Methods	Results (mm)	Authors’ Conclusions
PRP					
2014	Kundu et al. [42]	Evaluation of the effects of platelet-rich plasma (PRP) and different implant surface topography on implant stability and bone levels around immediately loaded dental implants.	A total of 30 implants divided into Group 1 (without PRP) and Group 2 (where implants were placed after dipping in activated PRP). Implant stability was measured with Periotest.	A statistically significant difference was noted in implant stability with PRP at baseline.	PRP-treated implant surfaces resulted in improved implant stability and bone levels.
PRF					
2012	Zhang et al. [48]	Influence of PRF on bone regeneration in sinus augmentation combined with DBBM.	Eleven sinuses were divided into a test group (DBBM + PRF) and control group (DBBM as the sole grafting material).	After six months, similar composition and distribution were found in both of the groups.	No significant difference between the groups was found.
2011	Somonpieri [49]	Clinical and radiological evaluation of the application of L-PRF as a sole grafting material in lateral sinus elevation procedures with simultaneous implant placement.	Twenty-three lateral sinus elevations were performed on 20 patients with simultaneous implant placement.	Six months after surgery, all implants were clinically stable during abutment tightening. Maximum follow-up at six years. Vertical bone gain between 8.5 and 12 mm.	L-PRF as the sole filling material during simultaneous sinus lift and implantation seems to be a reliable surgical option, promoting natural bone regeneration.
2018	Aoki et al. [50]	Evaluation of the application of PRF as a sole grafting material in sinus lift procedures.	A total of 71 implants in 34 patients after 1–7 years’ follow-up time. Statistical models for implant survival and potential factors associated with implant loss.	Seven implants were lost, and the cumulative survival rate at seven years by implant-based and patient-based analyses was 85.5% and 85.7%, respectively. Mean residual bone height (RBH) 4.26 mm. Greater implant survival rate for RBH < 4 mm than RBH ≥ 4 mm.	Sinus floor elevation with PRF alone could be applied in cases of lower RBH. However, it should be performed carefully in cases of RBH < 4 mm.
2018	Olgun et al. [51]	Clinical, histological, and radiographic comparison between autologous titanium-prepared PRF (T-PRF) and allografts in sinus lifting procedures.	Ten sinuses were randomly assigned to T-PRF as the test group, and 8 were assigned to allografts as the control group.	The control group showed better radiological results (62% in volume, 53% in density, and 69% in height) than the T-PRF group. Newly formed bone ratios were 17.28 ± 2.53 and 16.58 ± 1.05 in the allograft and T-PRF groups, respectively. There was no difference between the test and control groups (*p* = 0.611) in terms of implant stability values.	T-PRF alone in sinus lifting procedures revealed successful clinical and histomorphometric results.
2012	Tatullo et al. [52]	Clinical and histological evaluation of PRF in combination with deproteinised bovine bone (Bio-Oss) compared to DBBM alone in sinus elevation procedures.	Seventy-two sinus lifts with subsequent implant insertions.	Histological results after 106 days revealed that adding of PRF resulted in the formation of lamellar bone tissue with an interposed, richly vascularised stroma.	PRF and piezosurgery reduced the healing time compared to the 150 days described in the literature, facilitating optimal bone regeneration. At 106 days, it was possible to achieve good primary stability of endosseous implants, although lacking functional loading.
2013	Khairy et al. [53]	Evaluation of bone quality in sinuses augmented with autogenous bone, with or without platelet-rich plasma (PRP) mix.	In group “I”, five maxillary sinus lifts with autogenous bone augmentation and implant insertion were performed six months after grafting. Ten maxillary sinus lifts with autogenous bone augmentation were mixed with PRP, with implant insertion at 4 or 6 months post-grafting in Group II.	Six months after implant placement, Group II showed significantly higher mean bone density (*p* = 0.041). Histomorphometric analysis revealed that Group I had the highest mean value, and was statistically significant (39.5 ± 7.4; *p* = 0.003).	PRP did not reveal any significant impact on bone quality at 3 months after placement. Bone density was improved after 6 months.
2018	Nizam et al. [54]	Evaluation of the effects of leukocyte- and platelet-rich fibrin (L-PRF) combined with deproteinised bovine bone mineral (DBBM) on bone regeneration in maxillary sinus augmentation.	Twenty-six maxillary sinus augmentation procedures were randomly divided into a test group (DBBM + L-PRF) and control group (DBBM alone in a split-mouth design).	No significant differences in the evaluated parameters were observed.	Both techniques were effective for maxillary sinus augmentation. After six months of healing, adding L-PRF to DBBM did not improve the amount of regenerated bone or the amount of the graft integrated under histological and histomorphometric evaluation.

## Data Availability

Not applicable.
